
JOURNAL INFORMATION
==============================
NLM Title Abbreviation: ScientificWorldJournal
Journal ID: ScientificWorldJournal
NLM Journal ID: 101131163
Journal ID: TSWJ

The Scientific World Journal

ISSN: 2356-6140
EISSN: 1537-744X
Publisher: Wiley

ARTICLE INFORMATION
==============================
PMCID: PMC6083998
PMID: 30147599
DOI: 10.1100/tsw.2001.214
Article ID: 164061
Article version: 1
Subjects: Short Report

Nitric Oxide Activates Apoptosis Signal - Regulating Kinase 1 (ASK 1) Forming S-Nitrosothiols with Reactive Thioredoxin SH-Groups. Nitric Oxide Synthase (NOS) Induction Mediated Inhibition of Poly-(ADP-RIBOSE)-Polymerase (PARP) and Protein Kinase C (PKC) Activities as well as Intranucleosomal DNA Fragmentation (IDF) Increase in the HEP-2 Cells

Sumbayev Vadim V. *
Yasinska Inna M.
Department of Biochemistry I. I. Mechnikov Odessa National University, per. Shampansky 2, Odessa 65058, Ukraine
*Corresponding author.
Publication date: 2001
Electronic publication date: 2001 Nov 14
Volume: 1
First page: 118
Last page: 118
Copyright: Copyright © 2001 Vadim V. Sumbayev and Inna M. Yasinska.
Copyright year: 2001
License: This is an open access article distributed under the Creative Commons Attribution License, which permits unrestricted use, distribution, and reproduction in any medium, provided the original work is properly cited.
License URL: https://creativecommons.org/licenses/by/3.0/

==============================
Miami *Nature Biotechnology* Short Reports
*TheScientificWorld* (2001) 1 (S3), 118SR
ISSN 1532-2246; DOI 10.1100/tsw.2001.214

### NITRIC OXIDE ACTIVATES APOPTOSIS SIGNAL - REGULATING KINASE 1 (ASK 1) FORMING S-NITROSOTHIOLS WITH REACTIVE THIOREDOXIN SH-GROUPS. NITRIC OXIDE SYNTHASE (NOS) INDUCTION MEDIATED INHIBITION OF POLY-(ADP-RIBOSE)-POLYMERASE (PARP) AND PROTEIN KINASE C (PKC) ACTIVITIES AS WELL AS INTRANUCLEOSOMAL DNA FRAGMENTATION (IDF) INCREASE IN THE HEP-2 CELLS

Vadim V. Sumbayev* and Inna M. Yasinska

Department of Biochemistry I. I. Mechnikov Odessa National University, per. Shampansky 2, Odessa 65058 Ukraine
* inna@farlep.net

**INTRODUCTION.** ASK 1 was recently identified as mitogen activated protein (MAP) kinase kinase kinase and activates p53, Jun-N-terminal kinase and stress activated protein kinases through MAP kinase kinase cascade phosphorylation. These factors cause the induction of apoptosis. Mammalian thioredoxin was established to be the direct inhibitor of ASK 1. It has been reported that superoxide activates ASK 1 forming sulfinic ions with thioredoxin SH-groups and making ASK 1 free. We assumed that nitric oxide (NO) may interact with thioredoxin SH-groups forming S-nitrosothiols. It was found that NO mediates apoptosis through p53 activation and mitochondrial dysfunction. The aim of this work was to study if nitric oxide activates ASK 1 forming S-nitrosothiols with reactive thioredoxin SH-groups and also the effects of the induction of NOS activity in the HEP-2 cells on PARP and PKC activities as well as IDF.

**METHODS.** The effects of NO (supplied by sodium nitroprusside) on thioredoxin were studied on homogenous thioredoxin preparations and on rat liver homogenates. The quantity of S-nitrosothiols [1] and ASK 1 activity [2] were studied in 5, 10, 20, 30 and 60 min after injection of sodium nitroprusside to the rat liver homogenates. Induction of NOS activity in HEP-2 cells was mediated by supplying of $\gamma$-interferon ($\gamma$-IFN, free or together with superoxide generator xanthine oxidase inhibitor allopurinol) to the cultivating medium. In 24 hours cells were washed and lysed by sodium dodecylsulfate. Xanthine oxidase [3], NOS, PARP and PKC activities as well as IDF [4] were studied in the cell lysates.

**RESULTS.** It was found that nitric oxide forms S-nitrosothiols with homogenous thioredoxin. In the rat liver homogenates incubated together with sodium nitroprusside the ASK 1 activity increased during all 60 minutes of incubation (Fig. 1). The increase of S-nitrosothiols concentration in these reaction systems correlated with the ASK 1 activity increase (Fig. 2). The activation of ASK 1 by nitric oxide makes possible to conclude that it causes the release of ASK 1 from thioredoxin. This conclusion is proved by the increase of the quantity of S-nitrosothiols in the reaction system during all 60 minutes of incubation. It was established that in the HEP-2 cells where the nitric oxide synthase was activated PARP and PKC activities were decreased. The increase of the IDF in these cells was observed. In the cell cultures where allopurinol was not injected and xanthine oxidase was activated too PARP and PKC activities were increased and the IDF decrease in these cells was observed (Table 1).

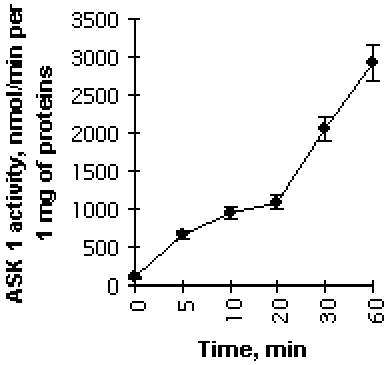

**Fig. 1**. Dependence of ASK 1 activity in the rat liver homogenate on the time of homogenate incubation with sodium nitroprusside.

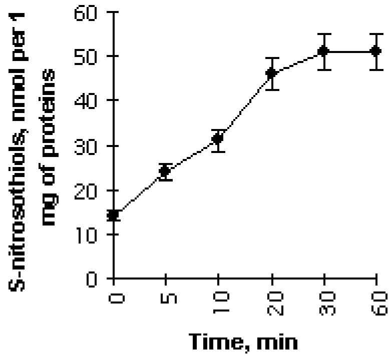

**Fig. 2**. Dependence of S-nitrosothiols concentration in the rat liver homogenate on the time of homogenate incubation with sodium nitroprusside.

**Table 1.** PARP and PKC activities as well IDF in the HEP-2 cells

| Cell group | PARP activity, μmol of poly-(ADP-ribose) per 1mg of protein | PKC activity, U/min per 1mg of protein | IDF, % |
|---|---|---|---|
| Control | 2.3 ± 0.2 | 194 ± 9 | 13.5 ± 0.3 |
| IFN-γ + allopurinol | 0.96 ± 0.1* | 162 ± 8* | 23 ± 0.8* |
| IFN-γ | 3.25 ± 0.25* | 226 ± 4* | 12.6 ± 0.2 |

**DISCUSSION.** Nitric oxide activates ASK 1 forming S-nitrosothiols with reactive thioredoxin SH-groups. Induction of NOS activity in the HEP-2 cells induces their apoptotic death. Both xanthine oxidase and NOS induction perform reverse effect that may be caused by peroxynitrite formation from NO and xanthine oxidase generated superoxide [4, 5]. Peroxynitrite was established to be cytoprotector that prevents to the apoptotic cell death.

**REFERENCES.**

1. Cook, J.A., Kim, S.Y., Teague, D., et al. (1996) Anal. Biochem. 238, 150-158
2. Sumbayev, V.V. and Yasinska I.M. (2000) LAMSO, 1, 95-100
3. Sumbayev, V.V. and Yasinska I.M. (2000) Exp. Oncol. (in press)
4. Sumbayev, V.V. (2000) Biochemistry (M) 65, 972-975
5. Bruene, B., Sandau, K., and von Knethen, A. (1998) Biochemistry (M) 63, 966-975
